# Peritumoral Fat Radiomics for Dual Prediction of TNM Stage and Histological Grade in Clear Cell Renal Cell Carcinoma: Discovery of Target-Specific Optimal Imaging Distances

**DOI:** 10.3390/diagnostics16071099

**Published:** 2026-04-05

**Authors:** Abdulrahman Al Mopti, Abdulsalam Alqahtani, Ali H. D. Alshehri, Ghulam Nabi

**Affiliations:** 1 Department of Radiological Sciences, College of Applied Medical Sciences, Najran University, Najran 55461, Saudi Arabia; analmopti@nu.edu.sa (A.A.M.); ahzafer@nu.edu.sa (A.H.D.A.); 2 Health Research Center, Najran University, Najran 55461, Saudi Arabia; 3 School of Medicine, Centre for Medical Engineering and Technology, University of Dundee, Dundee DD1 9SY, UK; gnabi@dundee.ac.uk

**Keywords:** clear cell renal cell carcinoma, radiomics, peritumoral microenvironment, TNM staging, WHO/ISUP grading, machine learning, computed tomography, tumor heterogeneity

## Abstract

**Background/Objectives:** Perirenal fat (PRF) constitutes a critical yet understudied component of the tumor microenvironment in clear cell renal cell carcinoma (ccRCC). While radiomics enables non-invasive tissue characterization, whether PRF-derived features can simultaneously predict both TNM stage and histological grade, and whether optimal peritumoral distances differ between these distinct biological targets, remains unexplored in the literature. **Methods:** This multi-cohort retrospective study included 474 histopathologically confirmed ccRCC patients from three independent datasets (2007–2023). Automated nnU-Net segmentation delineated tumors and kidneys. Concentric PRF regions were systematically generated at 1–10 mm radial distances, yielding 18 distinct regions of interest. From each ROI, 1409 radiomic features were extracted using PyRadiomics. Sequential feature selection employed correlation filtering, SHAP-guided elimination, and LASSO regularization. Multiple machine learning classifiers underwent hyperparameter optimization with rigorous cross-cohort validation. **Results:** Systematic ROI screening revealed target-specific optimal distances: 4 mm PRF for TNM staging versus 10 mm PRF for histological grading. For staging, the integrated model (tumor + PRF radiomics + clinical variables) achieved AUC 0.829 (95% CI 0.781–0.877), sensitivity 80.2%, and specificity 67.8%. For grading, the combined model achieved AUC 0.780 (95% CI 0.598–0.962), sensitivity 79.7%, and specificity 63.3%, significantly outperforming all single-compartment models (DeLong *p* < 0.001). **Conclusions:** This study establishes that PRF radiomics enables accurate simultaneous non-invasive prediction of both TNM stage and histological grade in ccRCC. The novel discovery that optimal peritumoral distances differ substantially by prediction target (4 mm versus 10 mm) suggests distinct biological underpinnings for stage- and grade-related microenvironmental alterations, with important methodological implications for radiomic model development in oncology.

## 1. Introduction

Clear cell renal cell carcinoma (ccRCC) represents the predominant histological subtype of kidney malignancies, accounting for approximately 75% of diagnoses and imposing substantial global morbidity with over 400,000 new cases annually worldwide [1,2]. Two pathological parameters fundamentally govern clinical management: Tumor, Node, Metastasis (TNM) stage, which guides surgical approach selection, systemic therapy decisions, and adjuvant treatment considerations; and World Health Organization/International Society of Urological Pathology (WHO/ISUP) histological grade, which informs long-term prognosis, surveillance intensity, and clinical trial eligibility [3,4]. The ability to accurately predict both parameters preoperatively would significantly enhance personalized treatment stratification, yet current practice relies on invasive tissue sampling through percutaneous biopsy or surgical resection, both carrying inherent limitations, including sampling heterogeneity and procedural morbidity.

The tumor microenvironment (TME) has emerged as a critical determinant of ccRCC behavior, extending beyond the tumor itself to encompass surrounding tissues that actively participate in neoplastic progression [5,6,7]. Perirenal fat (PRF), the adipose compartment immediately enveloping the kidney, occupies a privileged position at the tumor–microenvironment interface. Epidemiological investigations consistently identify obesity as an independent ccRCC risk factor, while mechanistic studies have elucidated how adipose tissue modulates tumor progression through complex adipokine signaling networks, inflammatory mediator secretion, and metabolic reprogramming [8,9]. Histopathologically, PRF invasion constitutes a critical staging criterion correlating with advanced TNM classification and diminished survival outcomes. Surgical series further demonstrate that adherent perirenal fat associates with aggressive tumor phenotypes and poses technical challenges during nephron-sparing procedures [10,11,12].

Radiomics offers a quantitative framework for high-throughput extraction of imaging features that capture tissue heterogeneity invisible to visual inspection, enabling non-invasive tissue characterization with potential for clinical translation [13,14,15]. Recent investigations have demonstrated the prognostic value of peritumoral radiomics across solid malignancies, with emerging evidence in ccRCC suggesting that the microenvironment surrounding tumors encodes clinically relevant biological information [16,17,18]. PRF compartments have increasingly been recognized as biologically active components of the ccRCC tumor microenvironment, with imaging signatures that may reflect tumor–fat interactions. In particular, Gill et al. demonstrated that contrast-enhanced CT texture features derived from juxtatumoral perinephric fat differ significantly between low- and high-grade ccRCC, supporting the premise that PRF encodes grade-related information beyond the tumor core [16]. More recently, Li et al. reported that radiomics derived from automatically segmented perirenal adipose tissue provides incremental value for pathological grading of ccRCC in a multicenter setting, reinforcing the clinical plausibility and scalability of fat-compartment radiomics [17]. Collectively, these studies motivate structured interrogation of tumor-adjacent PRF as a quantitative imaging biomarker with potential translational relevance.

Despite emerging signals, current evidence remains fragmented in ways that limit clinical translation. Many studies evaluate grade or stage in isolation, apply a single “juxtatumoral” or fixed peritumoral distance without testing whether the optimal distance depends on the biological endpoint, and often treat peritumoral tissue as a generic rim rather than isolating fat-specific perirenal compartments using adipose-appropriate intensity constraints. External validation across heterogeneous scanners and acquisition protocols is also inconsistently implemented, which complicates the interpretation of generalizability for perirenal fat radiomics and obscures whether target-specific peritumoral distances represent reproducible phenomena rather than dataset-specific artefacts.

To address these limitations, we present a multi-cohort framework that evaluates perirenal fat radiomics for simultaneous preoperative prediction of TNM stage and WHO/ISUP grade in ccRCC, using systematically constructed perirenal fat regions across multiple anatomically defined categories and radial distances. We further implement a rigorous cross-cohort validation strategy to test robustness under real-world acquisition heterogeneity and provide model interpretability analyses to characterize influential tumor and fat-derived radiomic signatures, thereby supporting both methodological transparency and biological interpretability. Reporting adheres to TITAN Guidelines 2025 for transparent artificial intelligence applications in medical research [19].

## 2. Materials and Methods

### 2.1. Study Design and Population

**Figure 1:**
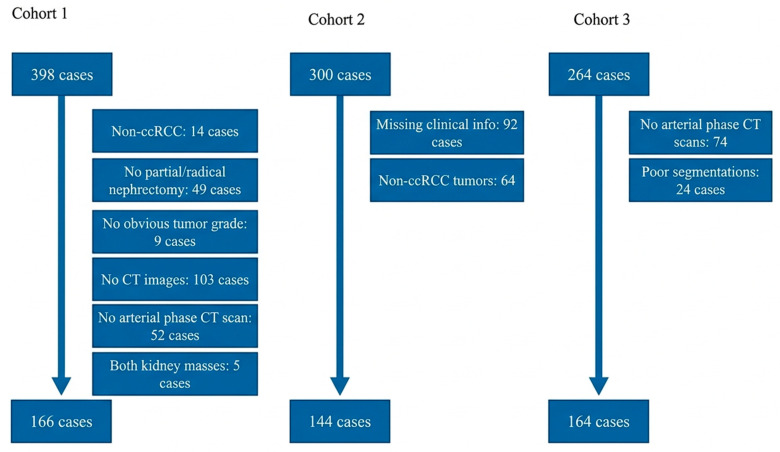
Patient selection flowchart. Consolidation of 962 initial ccRCC cases from three independent cohorts (institutional, KiTS19, TCGA-KIRC) yielded 474 patients meeting inclusion criteria for dual endpoint analysis (TNM staging and histological grading).

This retrospective multi-cohort study received institutional ethics approval (NHS Tayside reference IGTCAL12931) with waived informed consent given the retrospective design. The study population comprised 474 patients with histopathologically confirmed ccRCC diagnosed between 2007 and 2023, assembled from three independent sources representing diverse clinical settings and imaging protocols: Cohort 1 (institutional tertiary referral center; n = 166), Cohort 2 (KiTS19 challenge dataset; n = 144) [20], and Cohort 3 (TCGA-KIRC repository; n = 164) [21]. Inclusion criteria required the availability of arterial phase contrast-enhanced CT imaging with adequate perirenal tissue visualization and complete clinical documentation, including histopathologically confirmed TNM stage and WHO/ISUP grade (Figure 1). Patients were excluded for non-ccRCC histology, prior nephrectomy, incomplete staging or grading data, or inadequate image quality precluding reliable segmentation. TNM stage was dichotomized as low (stages I–II) versus high (stages III–IV), and histological grade as low (grades 1–2) versus high (grades 3–4) following established binary classification approaches in the radiomics literature.

### 2.2. Image Segmentation and ROI Generation

Native DICOM acquisitions underwent standardized preprocessing, including format conversion to NIFTI and region-of-interest cropping centered on the kidney with adequate peritumoral margin. Volumetric segmentation employed the nnU-Net deep learning framework pretrained on KiTS19 challenge data, representing a validated architecture for renal tumor delineation [22]. Because CT was acquired as a volumetric DICOM series, segmentation was performed in 3D on the resampled isotropic volumes. All automatically generated kidney and tumor masks were independently reviewed by two readers. Only a small number of cases required minor manual refinement (mainly boundary corrections), and the final segmentations were agreed by consensus before radiomic feature extraction. Since voxel-level ground-truth masks were not available across all cohorts, segmentation performance was assessed by expert review rather than Dice/Jaccard-based benchmarking. Post-segmentation processing included isotropic resampling to 1 × 1 × 1 mm^3^ resolution and Hounsfield unit normalization to standardize intensity distributions.

**Figure 2:**
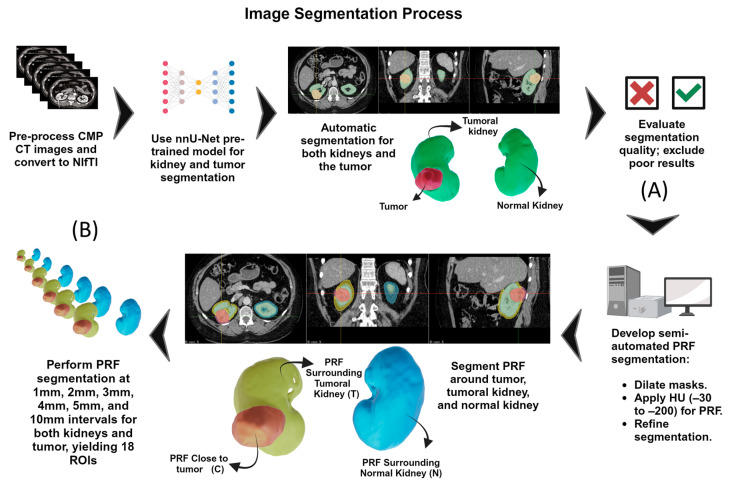
Automated segmentation pipeline. (**A**) Image preprocessing and nnU-Net segmentation of kidneys and tumors. (**B**) Generation of 18 concentric PRF regions at 1–10 mm radial distances.

PRF analysis incorporated three anatomically distinct categories to comprehensively evaluate the peritumoral microenvironment: PRF close to the tumor (PRF-C), PRF surrounding the entire tumoral kidney (PRF-T), and PRF adjacent to the contralateral kidney serving as an internal control (PRF-N). For each category, six concentric annular regions were algorithmically generated at radial distances of 1, 2, 3, 4, 5, and 10 mm from the respective boundaries, yielding 18 discrete PRF ROIs per patient. We selected 10 mm as the maximum distance to capture a biologically plausible peri-tumor adipose zone while limiting contamination from adjacent non-adipose structures and renal fascia; this range also aligns with common peritumoral radiomics distance selections in prior work. Adipose tissue isolation applied standardized HU intensity thresholding (−200 to −30 HU) to exclude non-adipose tissues. The complete segmentation workflow is illustrated in Figure 2; technical parameters appear in Supplementary Tables S1 and S2. Representative overlays illustrating segmentation and concentric PRF region generation are provided in Supplementary Figure S1.

#### Automated Segmentation: Architecture, Validation

Volumetric segmentation was performed using nnU-Net, a self-configuring deep learning framework built upon the U-Net encoder–decoder architecture [22], incorporating skip connections, instance normalization, and leaky rectified linear units as systematic defaults. The model was deployed using weights pre-trained on the KiTS19 challenge dataset [20] (210 annotated CT volumes) with the following training configuration: 1000 epochs; batch size 2; patch size 80 × 160 × 160 voxels; SGD optimizer with Nesterov momentum; combined cross-entropy and Dice loss with deep supervision; and extensive data augmentation [23]. No retraining was performed on the study cohorts.

On the independent KiTS19 held-out test set (n = 90), the nnU-Net achieved DSC 0.974, tumor DSC 0.851, and composite DSC 0.912, ranking first among 106 competing teams and outperforming the highest-ranked conventional 3D U-Net submission (kidney DSC 0.973, tumor DSC 0.825, composite 0.899) [23]. Full DSC and Jaccard values are provided in Supplementary Table S3. Segmentation accuracy across study cohorts was verified through systematic expert review, with manual correction required in fewer than 5% of cases.

### 2.3. Radiomic Feature Extraction and Selection

Quantitative feature extraction utilized the PyRadiomics platform following Image Biomarker Standardization Initiative guidelines and established CT radiomics protocols [24,25]. Feature computation incorporated 1409 features per ROI encompassing first-order statistics, shape descriptors, and texture matrices (GLCM, GLSZM, GLRLM, GLDM, NGTDM) with wavelet, Laplacian of Gaussian, and gradient filter transformations to characterize multi-resolution textural information. A three-stage sequential dimensionality reduction strategy addressed the high-dimensional feature space: (1) Pearson correlation filtering eliminated features with inter-correlation coefficients exceeding 0.9 to remove redundant information; (2) SHAP-informed recursive feature elimination with cross-validation identified discriminative feature subsets ranked by predictive importance [26]; (3) LASSO regularization with penalty parameter optimization performed final feature refinement. The SMOTE algorithm addressed class imbalance in training data [27]. Z-score normalization was applied independently to training and validation partitions to prevent data leakage. To avoid data leakage, all preprocessing and feature selection steps (correlation filtering, SHAP-guided elimination, LASSO, scaling, and SMOTE) were performed within the training partition only during model development; the locked feature set and fitted transforms were then applied to the held-out validation data.

### 2.4. Model Development and Statistical Analysis

Twelve machine learning classifiers were evaluated for each endpoint within a unified pipeline incorporating per-cohort SMOTE oversampling [27] and StandardScaler normalization, with all random states fixed at 42. To prevent data leakage, all preprocessing and feature selection steps, including correlation filtering, SHAP-based recursive elimination (ShapRFECV, step 0.5), LASSO regularization, z-score scaling, and SMOTE, were applied exclusively within the training partition of each outer fold; the held-out test cohort was not accessed at any stage of model development.

A nested cross-validation design was employed. In the inner loop, hyperparameter optimization (GridSearchCV) was performed using 10-fold stratified cross-validation within the training partition, with ROC-AUC as the optimization criterion. Full hyperparameter search spaces are provided in Supplementary Table S4. In the outer loop, generalization performance was evaluated using Leave-One-Cohort-Out (LOCO) cross-validation, in which each of the three independent cohorts (NHS Tayside, KiTS19, TCGA-KIRC) served as the held-out test set in rotation, yielding three test-fold results that constitute the complete output of this validation scheme.

Discrimination was quantified by the area under the receiver operating characteristic curve (AUC) with 95% confidence intervals derived from 1000 bootstrap iterations. Sensitivity and specificity were determined at the optimal operating point defined by the Youden index. Inter-model AUC comparisons were performed using the DeLong method [28]; statistical significance was set at *p* < 0.05. All analyses were conducted in Python (version 3.x) using scikit-learn, with SHAP employed for model interpretability [26]. A complete radiomics workflow is illustrated in the Supplementary Material Figure S2.

## 3. Results

### 3.1. Patient Characteristics

Following application of eligibility criteria, 474 patients were enrolled from the initial pool of 962 (Figure 1). Mean age ranged from 59.3 to 66.2 years across cohorts with consistent male predominance (58.4–65.2%). Body mass index data, available for Cohorts 1 and 2, demonstrated means of 30.1 and 31.4, respectively. High TNM stage (III–IV) was present in 27.1–44.0% of patients, while high histological grade (3–4) occurred in 31.9–59.8% across cohorts, reflecting the heterogeneous patient populations characteristic of multi-institutional studies (Table 1). Complete demographic and clinical characteristics, including stage and grade distributions, appear in Supplementary Table S5.

### 3.2. Discovery of Target-Specific Optimal Peritumoral Distances

**Figure 3:**
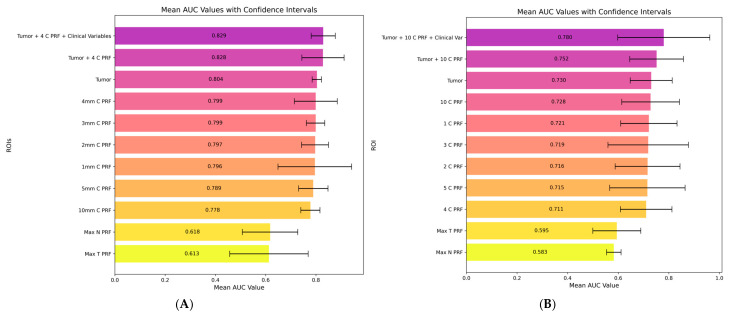
Target-specific optimal peritumoral distances. Maximum mean AUC values across ROIs for (**A**) TNM staging, 4 mm PRF-C optimal, and (**B**) histological grading, 10 mm PRF-C optimal. PRF-C: perirenal fat close to tumor; PRF-T: surrounding tumoral kidney; PRF-N: adjacent to contralateral kidney.

Systematic evaluation of radiomic models across all 18 PRF ROIs revealed a central finding of this investigation: optimal peritumoral distances differed substantially by prediction target (Figure 3). For TNM staging, the 4 mm PRF-C layer achieved maximum discrimination among PRF regions with a validation AUC of 0.799, comparable to tumor-derived features alone (AUC 0.804). In contrast, for histological grading, the 10 mm PRF-C layer demonstrated optimal performance with AUC 0.728, also comparable to tumor-only models (AUC 0.730). Models utilizing PRF-T and PRF-N consistently demonstrated limited predictive capacity for both endpoints (maximum AUCs 0.583–0.618), confirming that tumor-adjacent PRF (PRF-C) specifically encodes clinically relevant information. This target-specific distance pattern persisted across all classifier architectures evaluated, suggesting a robust biological phenomenon rather than an algorithmic artifact. Consistent performance across all three held-out cohorts confirmed the generalizability of these findings; per-fold AUC, sensitivity, and specificity values for each cohort are detailed in Supplementary Table S6.

### 3.3. Integrated Model Performance

**Figure 4:**
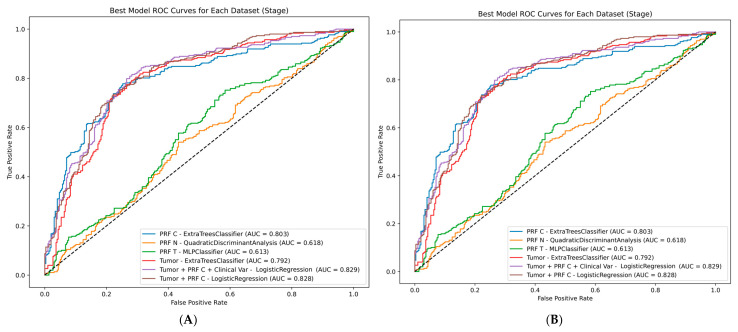
Receiver operating characteristic curves for (**A**) TNM staging models and (**B**) histological grading models (color-coded). Combined models incorporating tumor radiomics, target-specific PRF features (4 mm for staging, 10 mm for grading), and clinical variables achieved optimal performance for both endpoints.

For TNM staging, integration of tumor and 4 mm PRF-C radiomics improved discrimination to AUC 0.828 using logistic regression (sensitivity 80.9%; specificity 66.1%). Incorporating clinical variables (age, sex, BMI, tumor size) achieved optimal staging performance: AUC 0.829 (95% CI 0.781–0.877), sensitivity 80.2%, specificity 67.8%. Pairwise DeLong comparisons revealed no statistically significant differences among top-performing staging models (all *p* > 0.05), indicating that each model component contributed comparably to overall discrimination (Figure 4A; Table 2; Supplementary Table S7A).

For histological grading, a clinically important observation emerged: the tumor-only model achieved high sensitivity (87.2%) but poor specificity (43.1%), indicating systematic over-classification of high-grade disease that would translate to unnecessary aggressive management in clinical practice. Integration of tumor and 10 mm PRF-C radiomics using MLP improved balanced accuracy (AUC 0.752; sensitivity 81.9%; specificity 60.1%). The combined model incorporating clinical variables using QDA achieved optimal grading performance: AUC 0.780 (95% CI 0.598–0.962), sensitivity 79.7%, specificity 63.3%. Critically, DeLong tests demonstrated statistically significant superiority of the combined model over PRF-C alone (*p* = 0.0004), tumor-only (*p* = 0.0003), and combined radiomics without clinical variables (*p* = 0.0424), validating the additive value of multiparametric integration (Figure 4B; Table 2; Supplementary Table S7B).

SHAP analysis revealed distinct feature importance hierarchies between staging and grading models (Supplementary Figures S3 and S4). Staging models prioritized tumor shape metrics (least axis length: mean|SHAP| 0.100) and PRF texture features from the 4 mm zone (wavelet-hlh_glrlm_longrunemphasis: 0.060). Grading models weighted clinical TNM stage most heavily (0.140), followed by tumor morphology and 10 mm PRF texture heterogeneity features, with the larger peritumoral zone capturing grade-relevant microenvironmental alterations distinct from those informing staging.

## 4. Discussion

This multi-cohort investigation establishes PRF radiomics for simultaneous non-invasive prediction of both TNM stage and histological grade in ccRCC, while revealing a novel methodological finding: optimal peritumoral imaging distances differ substantially by prediction target. The 4 mm PRF-C zone proved optimal for staging (AUC 0.829), while the 10 mm layer excelled for grading (AUC 0.780), a distinction likely reflecting biologically distinct microenvironmental alterations associated with local invasion versus cellular dedifferentiation.

The target-specific optimal distance finding carries important methodological implications for the radiomics field. Prior peritumoral radiomics investigations typically employed fixed distances without systematic optimization [16,17,29]. Our results demonstrate that this “one-size-fits-all” approach sacrifices discriminatory power and that target-specific distance optimization should become standard practice. The smaller optimal distance for staging (4 mm) may reflect immediate peri-invasive alterations at the tumor–fat interface, consistent with the role of perirenal fat invasion in TNM upstaging, while the larger optimal distance for grading (10 mm) may capture broader adipose remodeling and inflammatory/metabolic signaling associated with aggressive tumor biology. Li et al. [18] similarly observed that 5 mm peritumoral regions outperformed 3 mm for grade prediction, consistent with our finding that more distant PRF captures grade-relevant information

Our staging results compare favorably with Demirjian et al. [30] (AUC 0.80) and Xia et al. [31] (AUC 0.82), while specifically interrogating the PRF adipose compartment with systematic distance optimization across multiple cohorts. For grading, our combined model’s balanced performance (sensitivity 79.7%, specificity 63.3%) addresses the clinically problematic over-prediction observed in tumor-only approaches (sensitivity 87.2%, specificity 43.1%). Wu et al. [32] demonstrated through radiogenomic analysis that radiomic signatures correlate with tumor microenvironment characteristics, providing biological plausibility for PRF-derived predictive capacity. The findings of this study are biologically plausible given evidence that perirenal adipose tissue participates in TME crosstalk, including inflammatory and metabolic signaling and adipose remodeling, which may influence tumor aggressiveness and invasion [33].

Clinical implications of accurate dual-endpoint prediction are substantial. TNM staging guides surgical approach selection and candidacy for systemic therapy, while histological grade informs long-term prognosis and surveillance intensity. Non-invasive prediction from routine preoperative CT could enhance personalized treatment planning while reducing reliance on biopsy procedures with inherent sampling limitations. Although the AUC gain over tumor-only models for staging was modest (0.829 vs. 0.804), PRF radiomics provides information that may improve risk stratification in borderline cases and reduce over-reliance on tumor morphology alone. For grading, integration improved specificity relative to tumor-only models, which is clinically relevant because reduced false high-grade classification could mitigate unnecessary aggressive management.

Study strengths include automated nnU-Net segmentation ensuring reproducibility, systematic evaluation of 18 peritumoral regions enabling target-specific distance discovery, and diverse multi-institutional cohorts enhancing generalizability. Limitations include retrospective design, imaging heterogeneity, and sample size constraints limiting subgroup analyses. Residual inter-scanner variability may affect radiomic feature stability; future work will include explicit harmonization (e.g., ComBat) sensitivity analyses and test–retest robustness where available. Future investigations should incorporate prospective validation and radiogenomic analyses exploring biological correlations of target-specific optimal distances.

## 5. Conclusions

This multi-cohort investigation demonstrates that CT-based peritumoral fat radiomics enables accurate simultaneous non-invasive prediction of both TNM stage (AUC 0.829) and histological grade (AUC 0.780) in ccRCC across three independent datasets totaling 474 patients. The discovery that optimal peritumoral imaging distances differ substantially by prediction target, 4 mm for staging versus 10 mm for grading, represents a methodologically important finding with broad implications for radiomic model optimization in oncological applications. These results support integrating peritumoral microenvironment assessment into preoperative ccRCC evaluation and establishing a framework for systematic target-specific distance optimization in peritumoral radiomics research. Prospective multi-institutional validation is warranted to confirm clinical utility and explore integration into routine clinical decision-making workflows.

## Figures and Tables

**Table 1:** Patient demographics and clinical characteristics across cohorts.

Variable	Cohort 1 (n = 166)	Cohort 2 (n = 144)	Cohort 3 (n = 164)
Age, mean (range)	66.2 (36.1–89.0)	59.3 (26.0–86.0)	60.3 (27.0–89.0)
Male, n (%)	97 (58.4%)	92 (63.9%)	107 (65.2%)
High Stage (III–IV)	73 (44.0%)	39 (27.1%)	68 (41.5%)
High Grade (3–4)	80 (48.2%)	46 (31.9%)	98 (59.8%)

**Table 2:** Performance metrics for optimal predictive models. AUC: area under ROC curve; CI: confidence interval; LR: logistic regression; ETC: extra trees classifier; QDA: quadratic discriminant analysis; MLP: multilayer perceptron.

Target	Model	Classifier	AUC (95% CI)	Sens	Spec
TNM Stage	Tumor + 4 mm PRF + Clinical	LR	0.829 (0.781–0.877)	80.2%	67.8%
	Tumor + 4 mm PRF	LR	0.828 (0.744–0.912)	80.9%	66.1%
	Tumor only	ETC	0.804 (0.785–0.822)	80.1%	70.5%
	4 mm PRF-C only	ETC	0.799 (0.714–0.885)	80.1%	69.0%
Histological Grade	Tumor + 10 mm PRF + Clinical	QDA	0.780 (0.598–0.962)	79.7%	63.3%
	Tumor + 10 mm PRF	MLP	0.752 (0.646–0.858)	81.9%	60.1%
	Tumor only	QDA	0.730 (0.647–0.814)	87.2%	43.1%
	10 mm PRF-C only	QDA	0.728 (0.614–0.842)	80.4%	53.0%

## Data Availability

The de-identified CT images and radiomic feature matrix derived from Cohort 1 (NHS Tayside) are not publicly deposited due to patient privacy regulations, but are available from the corresponding author (A.A.M.) upon reasonable request for non-commercial, academically led research with appropriate ethical approval. Cohort 2 (KiTS19) is publicly accessible at kits19.grand-challenge.org and Cohort 3 (TCGA-KIRC) is publicly accessible via The Cancer Imaging Archive at https://www.cancerimagingarchive.net/collection/tcga-kirc/ (accessed on 26 April 2024).

## Supplementary Materials

The following supporting information can be downloaded at: https://www.mdpi.com/article/10.3390/diagnostics16071099/s1, Table S1: CT Imaging Protocol Parameters; Table S2: PyRadiomics Feature Extraction Parameters; Table S3: nnU-Net and U-Net Segmentation Metrics on KiTS19; Table S4: Classifier Hyperparameter Search Spaces; Table S5: Extended Patient Demographics; Table S6: Per-Fold Cross-Validation Performance; Table S7: DeLong Test Pairwise Comparisons; Figure S1: PRF Region Segmentation Example; Figure S2: Radiomics Analysis Workflow; Figure S3: SHAP Feature Importance for TNM Staging Model; Figure S4: SHAP Feature Importance for Histological Grading Model.
