# Supplementary material for: Peritumoral Fat Radiomics for Dual Prediction of TNM Stage and Histological Grade in Clear Cell Renal Cell Carcinoma: Discovery of Target-Specific Optimal Imaging Distances
Source: Diagnostics (Basel). 2026 Apr 5;16(7):1099. doi: 10.3390/diagnostics16071099 (PMC13073437; doi:10.3390/diagnostics16071099)
Supplement: Supplementary file 1 [file diagnostics-16-01099-s001.zip › diagnostics-4164611-supplementary.pdf]

SUPPLEMENTARY MATERIALS

Peritumoral Fat Radiomics for Dual Prediction of TNM Stage and Histological Grade in Clear Cell Renal Cell Carcinoma: Discovery of Target-Specific Optimal Imaging Distances

Table S1. CT Imaging Protocol Parameters

Parameter	Cohort 1	Cohorts 2 & 3
Scanner	GE Healthcare (Helical)	Not specified
Slice Thickness	1.25 mm	Not specified
Tube Voltage	120 kVp	Not specified
Tube Current	Modulated	Not specified
Contrast Agent	Omnipaque 300 (Non-ionic)	Not specified
Injection Rate	3 mL/s	Not specified
Imaging Phase	Arterial	Arterial
Post-injection Delay	25–70 seconds	20–80 seconds

Table S2. PyRadiomics Feature Extraction Parameters

Parameter Category	Setting	Value	Description
Image Processing	resampledPixelSpacing	[1,1,1]	Isotropic voxel resampling (mm)
	interpolator	sitkBSpline	B-spline interpolation
	normalize	True	Enable normalization
	normalizeScale	500	Normalization scale factor
	removeOutliers	1.5	Outlier removal threshold (σ)
Feature Computation	binWidth	25	Fixed bin width (HU)
	force2D	False	3D feature computation
Filter Parameters	wavelets	coif1	Wavelet filter type
	logSigma	[3.0, 5.0]	LoG filter sigma values

Enabled Filters: Wavelet, Square/SquareRoot, Logarithm/Exponential, Gradient, Local Binary Pattern (LBP2D)

Table S3. nnU-Net and U-Net Segmentation Metrics on KiTS19

Model	Kidney Dice	Tumor Dice	Composite Dice	Kidney Jaccard	Tumor Jaccard
**nnU-Net (used in our study)**	**0.974**	**0.851**	**0.912**	**0.949**	**0.740**
**Standard 3D U-Net (Ma et al., KiTS19 5th place)**	**0.973**	**0.825**	**0.899**	**0.947**	**0.702**
**U-Net 2D (da Cruz et al.)**	**0.930**	—	—	—	—

Jaccard index derived as: Jaccard = Dice / (2 − Dice) as reported by Heller N, et al. 2021 [22].

Table 4. Classifier Hyperparameter Search Spaces

Classifier	Hyperparameter Search Space	Fixed Settings
Logistic Regression	C ∈ {0.001, 0.01, 0.1, 1, 10}	max_iter=1000, class_weight='balanced'
K-Nearest Neighbours	n_neighbors ∈ {3, 5, 7, 9}	—
Decision Tree	max_depth ∈ {3, 5, 7, 10}	class_weight='balanced'
Random Forest	n_estimators ∈ {100, 150, 200, 250}	class_weight='balanced'
LightGBM	n_estimators ∈ {100, 200, 300}	—
CatBoost	n_estimators ∈ {100, 200, 300}	silent=True
SVM	C ∈ {0.001, 0.01, 0.1, 1, 10}; γ ∈ {scale, auto, 0.1, 1, 10}	probability=True
MLP	solver ∈ {lbfgs, sgd, adam}; hidden_layer_sizes ∈ {(100,), (100,100), (200,200), (300,300)}; activation ∈ {relu, tanh, logistic}	max_iter=500, tol=1e-3
AdaBoost	n_estimators ∈ {50, 100, 200, 300}	—
Extra Trees	n_estimators ∈ {100, 200, 300, 400}; max_depth ∈ {3, 5, 7, 10}	—
QDA	reg_param ∈ {0.0, 0.1, 0.5, 1.0}	—
Gradient Boosting	learning_rate ∈ {0.001, 0.01, 0.1, 0.5, 1}; max_depth ∈ {3, 5, 7, 9}; n_estimators ∈ {100, 150, 200, 250}	—

Table S5. Extended Patient Demographics

Characteristic	Cohort 1 (n=166)	Cohort 2 (n=144)	Cohort 3 (n=164)
Age, mean (range)	66.2 (36.1-89.0)	59.3 (26.0-86.0)	60.3 (27.0-89.0)
Male, n (%)	97 (58.4%)	92 (63.9%)	107 (65.2%)
BMI, mean	30.1	31.4	N/A
Tumour size, mean cm	5.2	4.6	N/A
Stage I	83 (50.0%)	99 (68.8%)	81 (49.4%)
Stage II	10 (6.0%)	6 (4.2%)	16 (9.8%)
Stage III	71 (42.8%)	22 (15.3%)	43 (26.2%)
Stage IV	2 (1.2%)	17 (11.8%)	24 (14.6%)
Grade 1	11 (6.6%)	19 (13.2%)	1 (0.6%)
Grade 2	69 (41.6%)	80 (55.6%)	64 (39.0%)
Grade 3	63 (38.0%)	35 (24.3%)	72 (43.9%)
Grade 4	17 (10.2%)	11 (7.6%)	27 (16.5%)

Table S6. Per-Fold Cross-Validation Performance

Target	Fold	F1 (Class 0)	F1 (Class 1)	AUC	Sens	Spec
TNM Stage (LR)	1	0.777	0.814	0.852	0.887	0.708
	2	0.623	0.756	0.802	0.918	0.490
	3	0.761	0.687	0.826	0.596	0.862
Grade (QDA)	1	0.653	0.663	0.718	0.674	0.643
	2	0.767	0.824	0.883	0.938	0.660
	3	0.654	0.713	0.739	0.779	0.593

Table S7. DeLong Test Pairwise Comparisons

A. TNM Staging Models

Model 1	Model 2	AUC 1	AUC 2	P-value
Combined + Clinical	4mm PRF-C	0.829	0.799	0.4524
Combined + Clinical	Tumour only	0.829	0.804	0.2615
4mm PRF-C	Tumour only	0.799	0.804	0.7103

B. Histological Grading Models (*p<0.05)

Model 1	Model 2	AUC 1	AUC 2	P-value
Combined + Clinical	10mm PRF-C	0.780	0.728	0.0004*
Combined + Clinical	Tumour only	0.780	0.730	0.0003*
Combined + Clinical	Combined (no clinical)	0.780	0.752	0.0424*

Figure S1. PRF Region Segmentation Example

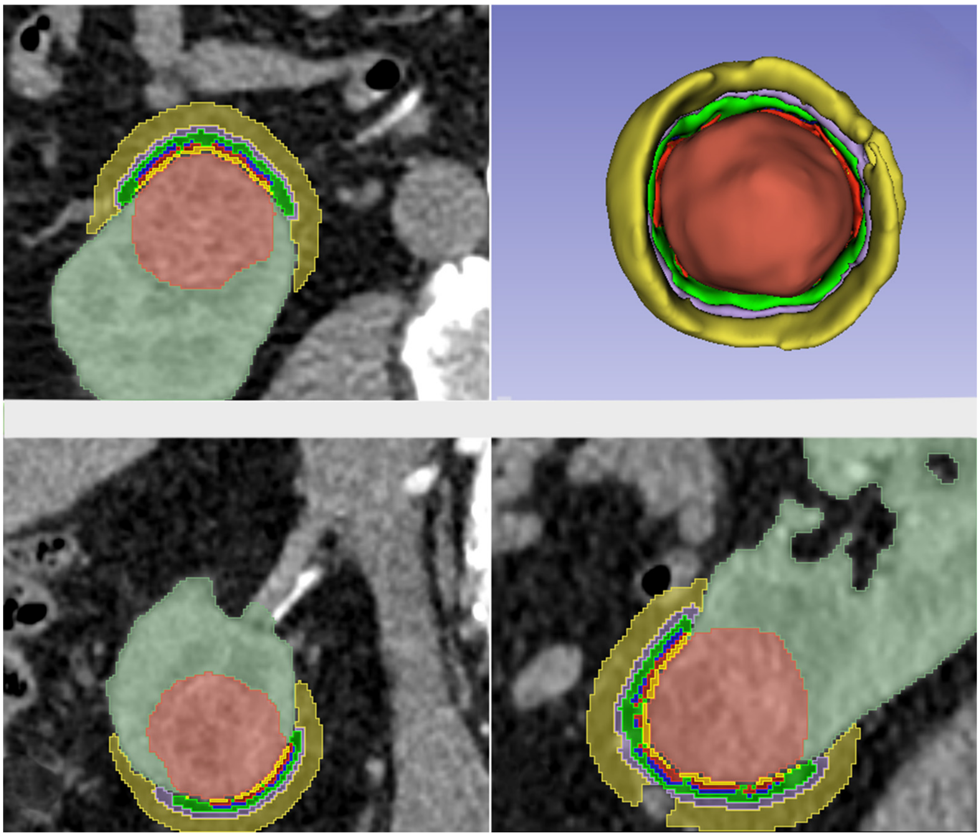

Figure S1: Representative CT imaging of ccRCC case demonstrating PRF ROI segmentation methodology. Axial (top left), coronal (bottom left), and sagittal (bottom right) views with 3D visualization (top right) of tumour ROI and six concentric PRF regions at 1-5 mm and 10 mm radial distances from tumour boundary. Colour-coded layers illustrate the systematic approach to peritumoral analysis enabling discovery of target-specific optimal distances.

Figure S2. Radiomics Analysis Workflow

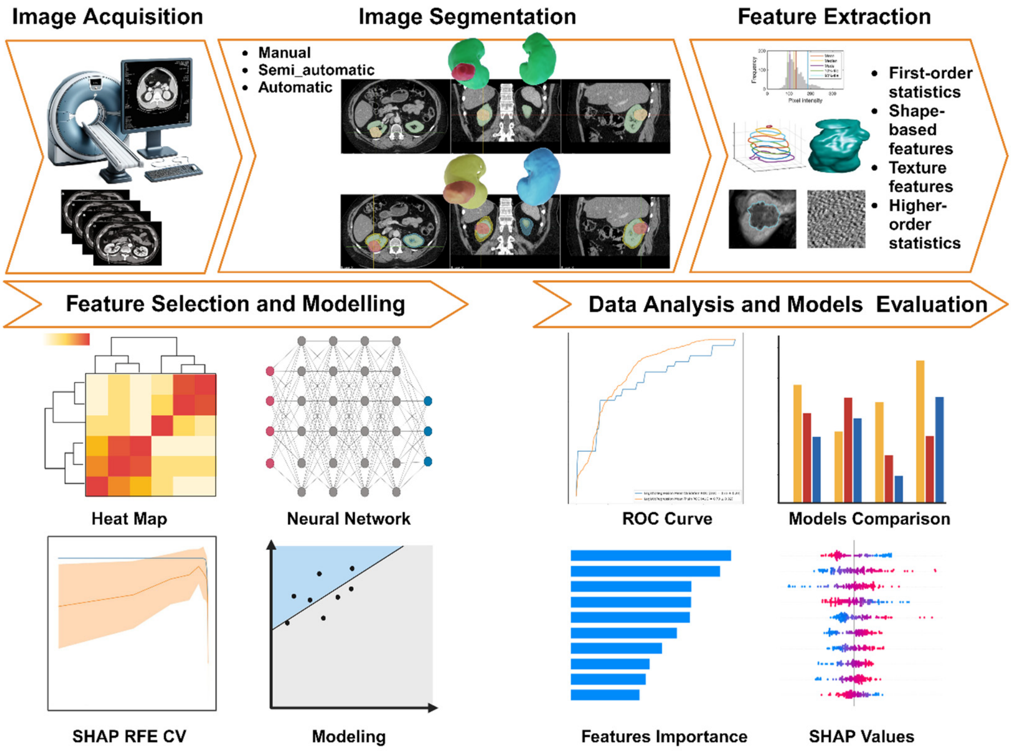

Figure S2: Complete radiomics workflow from image acquisition through feature extraction and dual-endpoint model development. Pipeline includes: (1) DICOM to NIFTI conversion, (2) nnU-Net automated segmentation, (3) PRF ROI generation at 18 distinct regions, (4) PyRadiomics feature extraction (1,409 features/ROI), (5) three-stage feature selection, and (6) machine learning model optimization with cross-cohort validation for both TNM staging and histological grading endpoints.

Figure S3. SHAP Feature Importance for TNM Staging Model

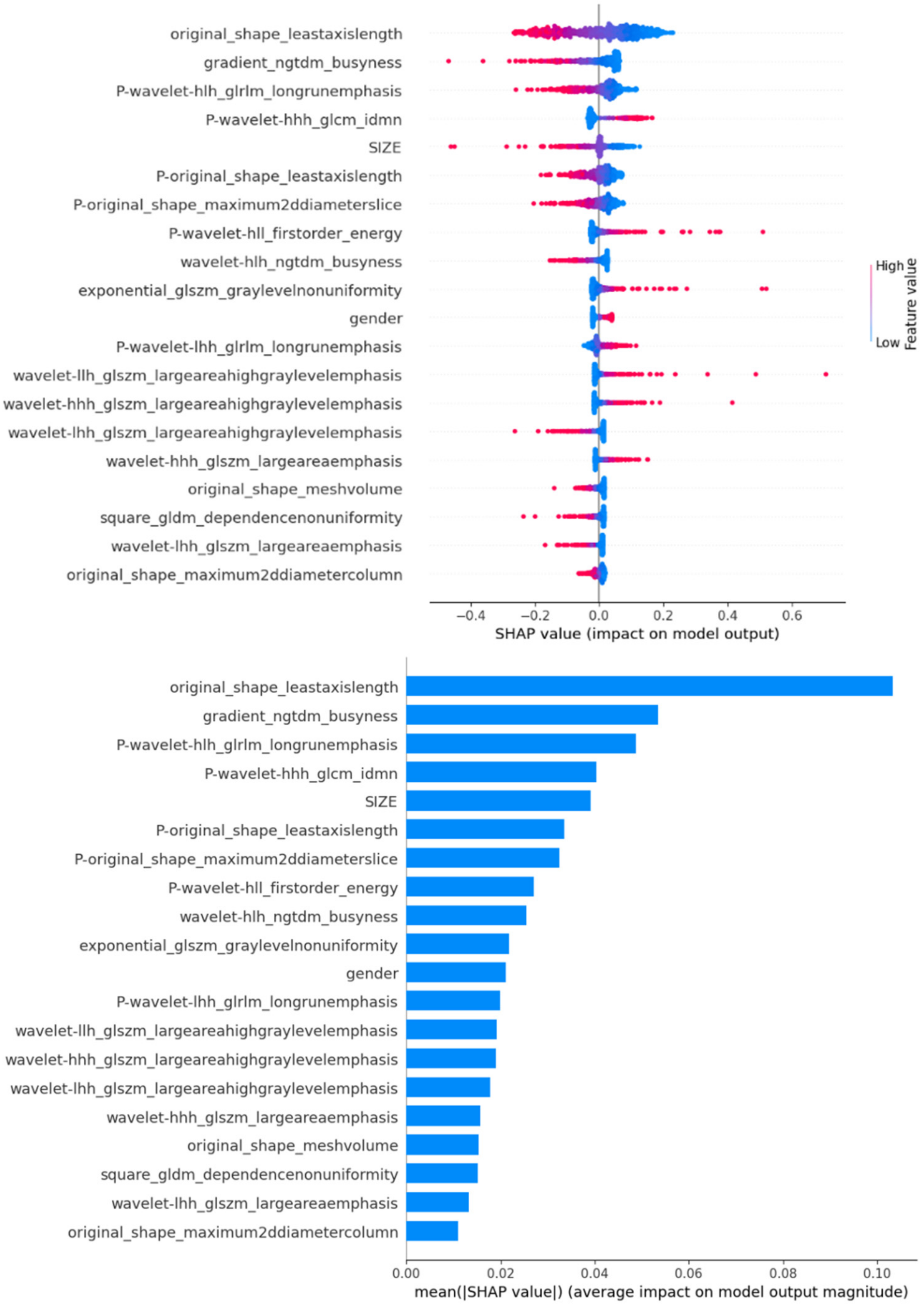

Figure S3: SHAP analysis for TNM staging model. (A) Summary plot showing feature impact distribution on model output. (B) Mean importance plot. Features prefixed with "P-" derived from 4mm perirenal fat region. Top contributors include tumour shape metrics (least axis length: 0.100 mean|SHAP|), PRF texture features (wavelet-hlh_glrlm_longrunemphasis: 0.060), and clinical variables (SIZE: 0.050).

Figure S4. SHAP Feature Importance for Histological Grading Model

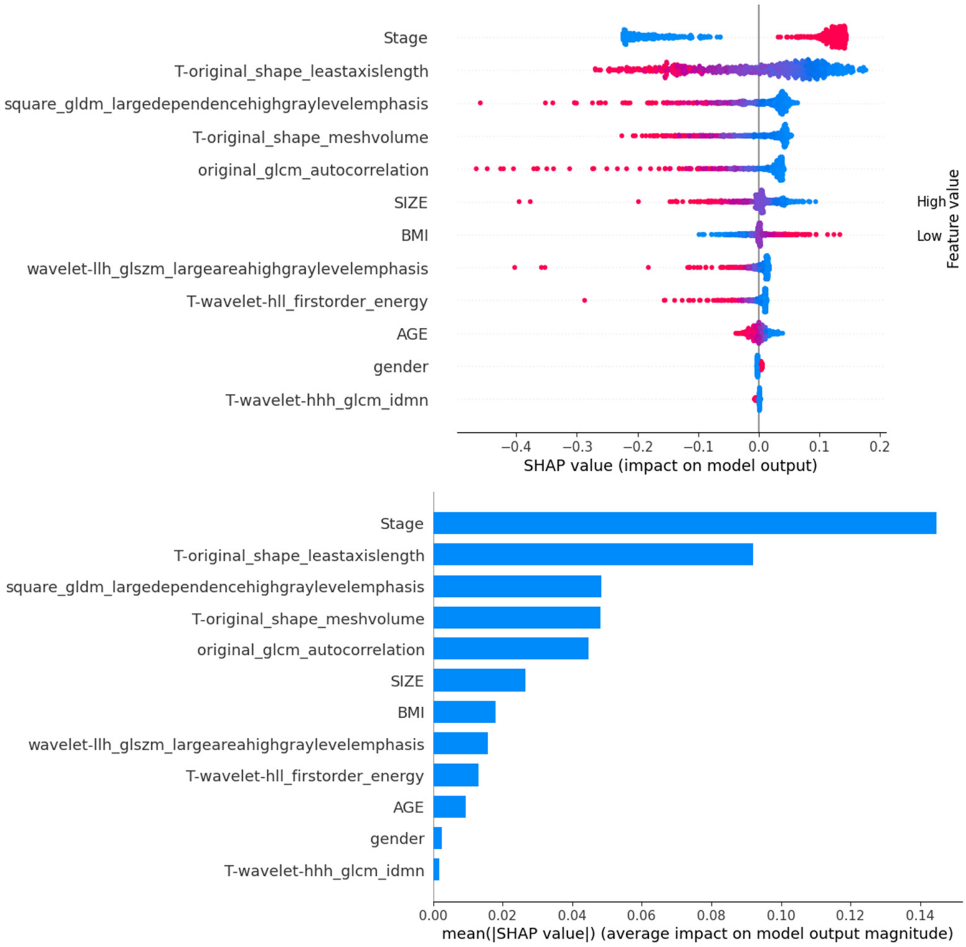

Figure S4: SHAP analysis for histological grading model. (A) Summary plot. (B) Mean importance plot. Features prefixed with "T-" derived from tumour region. Clinical Stage emerges as the dominant predictor (0.140 mean|SHAP|), followed by tumour shape (T-original_shape_leastaxislength: 0.110), and 10mm PRF texture features (square_gldm_largedependencehighgraylevelemphasis: 0.065).
